# Biodiesel Production Using Wild Apricot (*Prunus aitchisonii*) Seed Oil via Heterogeneous Catalysts

**DOI:** 10.3390/molecules27154752

**Published:** 2022-07-25

**Authors:** Batool Nisa, Fazal Ullah, Iqbal Nisa, Mushtaq Ahmad, Muhammad Zafar, Mamoona Munir, Shazia Sultana, Wajid Zaman, Hakim Manghwar, Farman Ullah, Muhammad Nauman Khan, Diaa O. El-Ansary, Hosam O. Elansary

**Affiliations:** 1 Department of Plant Sciences, Quaid-i-Azam University, Islamabad 45320, Pakistan; batoolnisa@bs.qau.edu.pk (B.N.); zafar@qau.edu.pk (M.Z.); mamoona_munir87@yahoo.com (M.M.); shaziaflora@hotmail.com (S.S.); 2 CAS Key Laboratory of Mountain Ecological Restoration and Bioresource Utilization, Chengdu Institute of Biology, Chinese Academy of Sciences, Chengdu 610041, China; fazal.ullah@bs.qau.edu.pk; 3 University of Chinese Academy of Sciences, Beijing 100049, China; 4 Department of Chemistry, Quaid-i-Azam University, Islamabad 45320, Pakistan; inisa@chem.qau.edu.pk; 5 Department of Botany, Rawalpindi Women University, Rawalpindi 46300, Pakistan; 6 Department of Life Sciences, Yeungnam University, Gyeongsan 38541, Korea; 7 Lushan Botanical Garden, Chinese Academy of Sciences, Jiujiang 332000, China; hakim@lsbg.cn; 8 Department of Forest Resources Management, College of Forestry, Nanjing Forestry University, Nanjing 210037, China; farman@bs.qau.edu.pk; 9 Department of Botany, Islamia College, Peshawar 25120, Pakistan; nomiflora@uop.edu.pk; 10 Biology Laboratory, Agriculture University Public School and College (AUPS & C), Boys, The University of Agriculture, Peshawar 25000, Pakistan; 11 Precision Agriculture Laboratory, Department of Pomology, Faculty of Agriculture (El-Shatby), Alexandria University, Alexandria 21545, Egypt; diaa.elansary@alexu.edu.eg; 12 Plant Production Department, College of Food and Agriculture Sciences, King Saud University, Riyadh 11451, Saudi Arabia; helansary@ksu.edu.sa

**Keywords:** biodiesel, nano-catalysts, heterogeneous catalyst, *Prunus aitchisonii* seed, oil cake

## Abstract

We confined the formation and characterization of heterogenous nano-catalysts and then used them to produce biodiesel from the novel non-edible seed oil of *Prunus aitchisonii*. *P. aitchisonii* seeds’ oil content was extracted at about 52.4 ± 3% with 0.77% FFA. Three different heterogenous nano-catalysts—calcined (CPC), KPC, and KOH-activated *P. aitchisonii* cake Titanium Dioxide (TiO_2_)—were synthesized using calcination and precipitation methods. The mentioned catalysts were characterized through XRD, SEM, and EDX to inspect their crystallin dimension, shape, and arrangement. Titanium dioxide has morphological dimensions so that the average particle size ranges from 49–60 nm. The result shows that the crystal structure of TiO_2_ is tetragonal (Anatase). The surface morphology of CPC illustrated that the roughness of the surface was increased after calcination, many macropores and hollow cavities appeared, and the external structure became very porous. These changes in morphology may increase the catalytic efficiency of CPC than non-calcined *Prunus aitchisonii* oil cake. The fuel belonging to PAOB stood according to the series suggested by ASTM criteria. All the characterization reports that *P. aitchisonii* is a novel and efficient potential source of biodiesel as a green energy source.

## 1. Introduction

*Prunus aitchisonii* belongs to the family Rosaceae, and is a unique potential non-edible (seed) oil-producing species and novel feedstock to produce biodiesel. A significant quantity of facts on the potential of coming generation of biofuels has been generated, and non-edible fruits used for biodiesel production include: *Pongamia pinnata* [1], *Jatropha curcas* [2], *Nicotiana tobacum* [3], *Hevea brasiliensis* [4] *Azadirachta indica* [5], *Silybum marianum* [6], *Camelina sativa* [7], *Eruca sativa* [8], and *Silybum eburneum* [9]. Chemical biodiesels are fatty acids methyl esters (FAMEs), having an extensive sequence of fatty acids comprised to mono-alkyl esters, extracted using plant feedstock and also a suitable choice of fuel [10]. Biodiesel has various advantages over conventional diesel, including high flash point, lubricity, bio-degradability, and reduced formation of pollutants when burned [11,12]. On the other hand, there are key shortcomings are associated with biodiesel production, such as high energy consumption, scaled-up expenditures, the food controversy debate, and expensive raw materials. Furthermore, the plodding reduction of traditional Petro diesel resources in the current era of greater consumption and rising energy demand, is a concern promoted via the “peak oil” theory. According to this theory, the greater demand for oil will surpass the available resources; the difference between the demand and resources will rise continuously [13]. It is crucial to seal this gap to explore the oil potential of apposite non-edible fruit such as *P. aitchisonii*. Non-edible seed-oil production has been considerably enhanced to explore whether biodiesel possesses the possible influence to solve the problems related to energy crises and a new way to produce green energy sources.

Usually, methanol and suitable catalysts are recycled for the synthesis of biodiesel through the transesterification of seed oil [14]. Conventional homo-catalysts are broadly used for industrial-level production [15]. Though they offer a higher yield, numerous demerits are coupled with higher production, i.e., the requirement of excess methanol [16], steps of neutralization that require considerable time and effort, and washing and drying steps to remove glycerol and catalysts which increases the cost of the product and also wastage of water [17,18]. Due to their possible part in challenging the disadvantages of standardized homogeneous catalysts, heterogeneous catalysts are receiving much interest for synthesizing biodiesel. However, this is slowly and gradually changing to the industrial level [19]. To fulfill these challenges, heterogenous nano-catalysts have several advantages such as higher conversion rate, high reactant productivity, substantial surface area, higher catalytic efficiency, better rigidity, higher resistance, and high waterproof property to saponification [20,21,22]. Additionally, these nano-catalysts can combat environmental contamination whenever used as a cost-effective fuel. Most techniques have been practiced for synthesizing these mano-catalysts owing to their eco-friendly and non-innocuous nature [23]. Additionally, almost every part of the plant (stems, roots, seeds, latex, etc.) can be used to prepare these plant-based nano-catalysts. 

Among alkali and acid mixed solid catalysts, alkali ones offer higher proficiency over acid catalysts for biodiesel production [24]. The main benefit of heterogeneous catalyst utilization is that they are not soluble in biodiesel. These catalysts stay non-destructive with less maintenance and a lower cost of separation. A number of studies have been done on nano-catalysts, demonstrating the recent enthusiasm for transesterification with the application of nanotechnology [25]. Using heterogeneous nano-catalysts, the best information centered on the literature studied. The subject of various recently updated publications was both quantitative and qualitative analyses of FAMEs (Free Fatty Acids, Methyl Esters) and the nature of non-edible seed oils. However, no effort has been made to investigate the potential of *P. aitchisonii* seed oils to produce biodiesel. The present study on the seed oils of *P. aitchisonii* conducted complete investigations for the initial time and nominate them as an innovative supplement in non-edible feedstock to produce biodiesel by utilizing the relative application of three different mixed nano-catalysts (HCNs). The current research deals with the comparative use and application of three nano-sized (heterogenous) alkali catalysts (CPC, KPC, and TiO_2_) that are effective, cost-efficient, and provide the maximum benefit due to their recyclable nature. The comparative evaluation has been made in terms of their characterization of biodiesel production utilizing non-edible seed oil of *Prunus aitchisonii* such as X-ray Diffraction (XRD), SEM, (Scanning Electron Microscopy), EDX, (Energy-dispersive X-ray spectroscopy). Furthermore, quantitatively, the blended FAMEs were analyzed using systematic techniques, i.e., FT_IR (Fourier Infra-Red Spectroscopy), GC_MS (Gas Chromatography, Mass Spectrometry), and NMR (Carbon and Proton Nuclear, Magnetic Resonance).

## 2. Methodology

**Figure 1:**
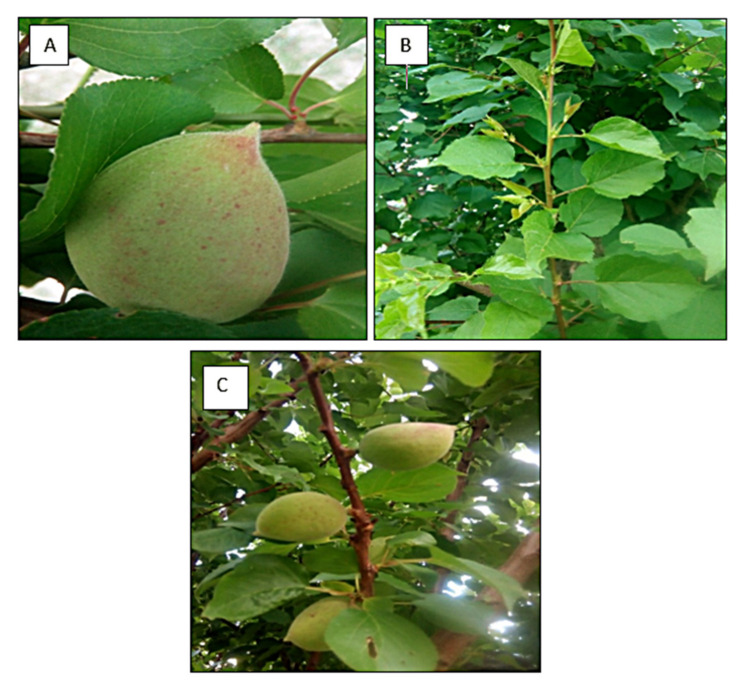
*Prunus aitchisonii* (**A**) fruit, (**B**) leaves, (**C**) fruits, leaves and branches.

The experimental work and subsequent analysis were carried out at the Biodiesel laboratory (Department of Plant Sciences, Quaid-i-Azam University Islamabad, Pakistan). Specimens for the present research work were collected from Parachinar, District Khuram Khyber Pakhtunkhwa, during the summer of 2018 (Figure 1). Seeds of *P. aitchisonii* plants were collected for biodiesel analysis. The collected seeds were properly washed with warm distilled (pure) water to eliminate dust. Then, they were dehydrated in an oven at 60 °C for 24 h. The organic solvent extraction (Soxhlet apparatus) process has been determined to check the percentage of oil from the seed of *P. aitchisonii*. An electric (oil) expeller was used to extract crude oil (Model, KEK P0015, 10127, Remscheid, Germany).

### 2.1. Equipment and Chemicals Used in Seed Oil Extraction

The types of equipment used are electric balance (Mod: GF-3000), thermometer, digital hotplate (Mod: AM4, VELF SCIENTIFICA, Usmate, Italy), beakers (100 mL and 500 mL), burette (100 mL), pipette (10, 15 mL), China dish, Teflon magnetic stirrer, pH meter, steel spatula, pestle, mortar, and furnace. 

Chemicals used were Chloroform (CHCl_3_), Titanium isopropoxide, n-hexane (C_6_H_14_), Petroleum ether, Sodium hydroxide (NaOH), Methanol (99.9% pure), Calcium carbonate, Potassium hydroxide (KOH), Ethanol absolute (Scharlau-99.8% pure), C_3_H_8_O (isopropyl alcohol), and distilled water is used. The chemicals used are of diagnostic grade delivered by Abbott, Merk, and Sigma Aldrich (chemical) laboratories.

### 2.2. Extraction of Oil

#### 2.2.1. Chemical Extraction of Oil

The organic solvent was used for the removal of chemical constituents from a sample, also termed solvent extraction. (Soxhlet) apparatus was used for this purpose. The dehydrated seeds are kept in a fine powder using a pestle and mortar. Petroleum ether filled up to 250 mL in a round-bottom flask in the Soxhlet apparatus. A thimble holding crushed powder of seeds was positioned in the center of the apparatus and heated up to 60 °C for about 5–6 h. In a round-bottom flask, the oil droplets were easily observable, and the solvent was constantly reused and recovered. The crushed wet sample was again dried via an oven to vaporize the solvent. The decrease in the sample’s weight determined the oil content in the seeds using Equation (1).

% age of (crude) oil W4 = [W3 − W1/W2] ×100(1)

W1 = empty flask weight, W2 = weight of powdered sample W3 = weight of oil and flask, W4 = oil’s weight

#### 2.2.2. Mechanical Extraction of Oils

**Figure 2:**
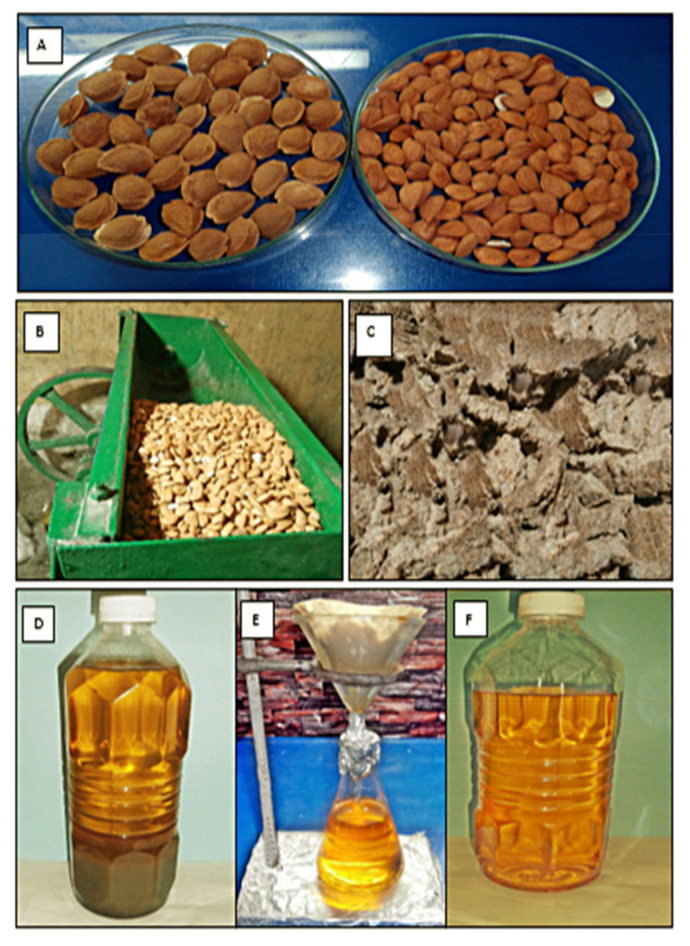
(**A**–**F**). (**A**) Shelled and deshelled seeds of *Prunus aitchisonii*. (**B**) Seeds in oil expeller. (**C**) Oil cake. (**D**) Crude oil. (**E**) Crude oil filtration. (**F**) Filtered pure oil.

Mechanical extraction is one of the collective methods of oil extraction from different seeds. We used 10 kg of *P. aitchisonii* seeds in the electric oil expeller of (KEK, P0015 10127, Remscheid, Germany) and collected crude oil in bottles for further analysis. Subsequently, oil was extracted from the entire collection of seeds in 4–5 attempts [26]. After extraction, the crude oil of *P. aitchisonii* contains a lot of impurities and suspended solid particles that could affect the quality as well as the yield of the product. Crude oil was filtered out using the method used in [27]. Afterward, sieved oil was kept for accompanying experimental processing in a close-fitting glass container and stockpiled at optimum temperature. The seeds, oil expeller, oil cake, crude oil filtration, and filtered pure oil are given in Figure 2. 

### 2.3. Calculation of Free-Fatty Acid (FFA) Contents of Prunus aitchisonii Seeds Oil

Two types of titrations were used to determine free fatty acid value in oil, i.e., blank titration and sample titration. About 0.14 g of KOH was dissolved in 100 mL purified water to prepare 0.025 M, KOH solution for blank titration and transferred into the burette. For the preparation of indicator solution, 0.5 gm phenolphthalein was mixed in 50% ethanol. In a conical flask, up to 10 mL isopropyl alcohol was poured, 2 drops of indicator solution were added, and then the mixture was titrated against KOH 0.025 M solution till the pink color of the solution appeared. KOH volume, which was used in titration, was noted at that point where a change in the color was observed, and the experimentation was repeated three times. The average mean was proposed from all the amounts of KOH solution used in the blank titration. 

For the sample titration, the procedure remains the same, but the difference was that here, 9 mL isopropyl alcohol, 1 mL oil, and 2 drops of the indicator were mixed in a prepared solution in a conical flask. The mixed solution was titrated under 0.025 M KOH solution in the burette till the pink color appeared. KOH volume was recorded by repeating the same experiments three times to calculate the mediocre volume of KOH consumed during titration. The following formula is used for the calculation of the acid value of oil.

Acid Number = Amount of KOH used up in sample titration − Volume of KOH used during blank titration × Quantity of catalyst in g/L ÷ Volume of oil used.(2)

### 2.4. Production of Heterogeneous Nano-Catalysts (HNC)

#### 2.4.1. Calcined *Prunus aitchisonii* Cake (CPC)

*P. aitchisonii* cake was washed extensively with tap water 3–4 times to remove dust particles, soil particles, and other dirt particles, and after that it was dipped in distilled water. Having cleaned and washed the cake, it was dried in the oven at 70 °C, awaiting the removal of all moisture content to obtain a constant weight. After that, the dried cake was crushed, ground in a pestle and mortar, and placed in a tube inside a muffle furnace for calcination at 450 °C for 3 h. Calcined CPC was crushed into a powdered form and preserved as a heterogeneous catalyst for transesterifying *P. aitchisonii* seed oil (PASO).

#### 2.4.2. KOH-Activated *P. aitchisonii* Cake (KPC)

*P. aitchisonii* cake was washed and oven-dried at 70 °C for moisture content removal. The dried cake was then mixed with KOH (1 M). The mixture of KOH and cake was heated (70 °C) at that point to transform it into a paste. As a result, the paste of KOH-activated *P.* *aitchisonii* cake was produced. This cake paste was placed in a muffle furnace for 3 h at 450 °C. The resulted KPC was ground and kept to be used as the heterogeneous catalyst for *P. aitchisonii* seed oil transesterification.

#### 2.4.3. Titanium Dioxide (TiO_2_) Nanoparticles through Precipitation Methods

All the chemical substances were of the systematic mark used in the experiment, commercially purchased from Merck without further purification. Titanium isopropoxide and isopropyl alcohol were used as a precursor for synthesizing TiO_2_ nanoparticles. About 100 mL of isopropyl alcohol was poured into 15 mL titanium isopropoxide, and stirred for about 30 min. Likewise, 0.1 g PVP (Polyvinylpyrrolidone) was mixed in a solution and stirred for about 20 min. Later, we added 10 mL of distilled water dropwise to the previous solution for hydrolysis. As a result, Ti (OH)_4_ was formed as a snowy precipitate that was then refluxed for about 2 h. We also continuously stirred keep for one day. Moreover, the resultant precipitate was centrifuged with ethanol and purified water to remove impurities. The purified precipitate was dried at 80 °C for 1 day after centrifuging. As a final point, the precipitates of Ti (OH)_4_ were transformed into TiO_2_ nanoparticles at about 800 °C. A significant change of Ti (OH)_4_ phase into TiO_2_ was found to be above 400 °C. On behalf of the approval of particle size, the prepared sample became ready in powdered form for further examination such as via SEM and XRD [28], and then used for transesterification.

### 2.5. Illustration of Heterogeneous Nanocatalysts (KPC, KPC, and TiO_2_)

#### 2.5.1. XRD (X-ray Diffraction)

XRD (Model No. D8 Advance Bruker) was used to characterize catalysts used in the present work to safeguard the establishment of a wanted crystal-like assembly of all nanoparticles. With the help of the Scherer equation, the calculation was completed, which provided a heterogeneous ordinary diameter of nanoparticles. All dimensions were achieved between 10–60 °C.

#### 2.5.2. Scanning Electron Microscopic Technique (SEM)

SEM and EDX were accomplished through SEM, (Model JEOL JSM-5910 Tokyo, Japan, & HT7800 Ruli, Hitachi, Tokyo, Japan), respectively. Scanned images were obtained through operating field emissions of S-E microscope with 20 kV accelerating voltage. It aided the interpretation of the phenomena that occurred during calcining and pre-treatment and permitted the qualitative characterization of the surface of catalysts. 

#### 2.5.3. (EDS or EDX) Energy-Dispersive X-ray Composition Analysis

The EDX (Energy-Dispersive X-rays) indicates visibly the presence of different elements in a catalyst that has been synthesized. This analysis was also achieved by using Model-JOEL (JSM-5910) and (HT7800 Ruli) microscope, respectively.

### 2.6. Biofuel/Diesel Production through Reflux (Trans-) Esterification Reaction

Trans-esterification was approved through the reflux method for biodiesel synthesis from *P.* *aitchisonii* seed oils. Trans-esterification is a reaction in which fat reaction occurred with alcohol (glycerol and esters). The reaction was carried out in 2 curved-bottom flasks (250 mL) fortified with a magnetic stirrer and a reflux condenser. The oil/methanol ratio was taken as 1:4 and 1:5 refluxed with 0.20–0.25 g of catalysts for about 50 min at 60 °C. We boiled the seed oil at 120 °C for about 2 h. After cooling the preheated seed oil to 60 °C at room temperature, we added this oil into two necked round-bottom flasks. Again, this was refluxed for about 2 h at 70 to 75 °C. Subsequently, the reaction was completed, then the mixture was poured into the separation funnel for phase partition. The superior layer is alkyl esters, while the inferior layer is glycerin. Purification of biodiesel was approved by eliminating additional methanol in the apparatus called rotary (rotavaps). The ultimate reaction yield was calculated with Equation (2).

(3) $$\%\;\mathrm{yield}=\frac{\mathrm{amount}\;\mathrm{of}\;\mathrm{biodiesel}\;\mathrm{produced}}{\mathrm{the}\;\mathrm{volume}\;\mathrm{of}\;\mathrm{oil}\;\mathrm{produced}}\times 100$$

### 2.7. Characterization of Manufactured Fatty Acids-Methyl Esters

#### 2.7.1. Gas Chromatography-MS Analysis

The conformation of fatty acids in *P. aitchisonii* oil biodiesel (PAOB) was determined through GC-MS, having a quadrupole mass spectrometer with a double inlet differential vacuum system. Helium gas is supplied as a carrier gas with a definite rate of “1.44 mL/min”. The column heat was automated between 120–250 °C with a flow rate of 80 °C/min. Moreover, injector hotness was kept at 120 °C, and that of the sensing element as standard was kept at 250 °C. The volume of PAOB taken as 0.1 µL that was already dissolved in CHCl_3_, was injected with a splitting ratio of 1:3 using split mode. The mass spectrograph was fixed to scan from 50 to 1000 *m*/*z* value with electron impact ionization. The overall analysis was continued for about 31 min. 

#### 2.7.2. Magnetic Resonance Spectroscopy (MRS) or Nuclear–Magnetic Resonance (NMR)

NMR is recognized as a reliable and quick analysis used for identifying biodiesel quality. Total methyl esters have been supplied. ^1^H NMR and ^13^C NMR examinations were used for the trans-esterification reaction monitoring. The (biodiesel) sample was categorized by ^1^H NMR spectroscopy via “Avan CE 300 MHz” mass spectrometer supplied with 5 mm probes at 7.05, T, and Tetra methyl-silane and Deuterated chloroform were recycled as the solvent and interior stock, respectively. At a duration of about (30°), a spectrum of ^1^H NMR (300 MHz) was recorded, with a recycle interval of 1.0–8 scans. However, the range of carbon ^13^C NMR (75 MHz) was documented with a pulse interval of 30° and a reprocess stay of 1.89–160 scans. For the quantification of trans-esterification yield, a subsequent calculation using Equation (4).

C = (100) × 2A_Me_/3A_CH2_ (4)

Here C = measurement of transformation of triglycerides to corresponding methyl-esters, (A_Me_) = methoxy protons integration value of biodiesel, also (A_CH2_) = value of alpha-methylene protons.

#### 2.7.3. FT-IR

FT-IR (Fourier Infra-Red Spectroscopy) is one of the scientific techniques used for the monitoring of structural composition and functional groups present in free fatty acid methyl ester (FAME) [29] of *Prunus aitchisonii* oil biodiesel (PAOB). By this technique, the analysis of the structural composition of (PAOB) samples through Exc. Model FTS300MX (Bio-Rad-Excalibur CA, USA), ranging from 400–4000 cm^−1^. The tenacity was 1–15 scans for PAOB scrutiny.

#### 2.7.4. Methyl Esters Physical-Fuel Properties

Tests for fuel properties of *P. aitchisonii* were done by the Pakistan State Oil Company LTD, Central Laboratory KTA. These were compared with international standards issued by the Society of America for Materials Testing (ASTM) for quality guarantee. The physical properties of fatty acid/methyl ester (FAMEs) include the following: Color, Flashpoint (PMCC), Density, @ (15 °C) kg/L, Kinematic-viscosity, @ 40 °C, Pour point °C, Cloud point, Sulfur contents (% weight), (TAN; total acid number, mg KOH/gm). All agreed with ASTM D-1500, D-93, D-1298, D-445, D-97, D-2500, D-4294, D-974 correspondingly.

## 3. Results and Discussion

### 3.1. Biofuel Production of Seeds of Prunus aitchisonii

*P. aitchisonii* seeds oil (PASO) to biodiesel both definite values of oil and free-fatty acid (FFA) contents of seeds were calculated accurately. Thus, the oil content obtained on dry biomass was noted as (52.4 ± 3%), which is significantly innovative from all other non-edible and edible sources of seed oil, i.e., *Silybum marianum* (26.14%) [30] *Silybum eburneum* (37.7%) [9] *Citrus reticulate* (28.5%) [31], *Glycine max* (18–22%) [32,33], *Eruca sativa* (35%) [8], and *Capparis spinosa* [34]. However, for F.F.A of seed oil at 0.76 mg, KOH/g was verified. Quality, as well as biodiesel yield, were extremely dependent upon feedstock value. Mainly, the FFA content directly relates to the volume of biodiesel produced. Agreeing with the literature, the most appropriate FFA threshold of crude oils for efficient conversion into biodiesel was measured up to 3% [35,36] but beyond this limit, proficiency decreases gradually, and indications of the course called saponification (formation of soap), which makes the departure process tougher [37,38]. For maximum conversion of PASO into biodiesel, three heterogeneous catalysts were employed with different concentrations (*w*/*w*), i.e., CPC, KPC, and TiO_2_. Calculations granted in (Table 1) significantly demonstrate the dependence of oil conversion rate on the kind and quantity of catalysts, oil versus methanol ratio, rate of temperature, and the period during the trans-esterification reaction. At 0.25 gm and 0.20 gm of mentioned catalysts loaded, the alteration percentage is reasonably greater, about 96.5% for TiO_2_ and 94.5% and 92.58% KPC and CPC, respectively. Similarly, at the specific temperature 70 °C, CPC and KPC show their highest conversion while that of TiO_2_ was at 75 °C, while other variables remain constant. However, it is also clear from (Table 1) that at a 1:5 oil to methanol ratio, the highest conversion occurs through both CPC and KPC, while for TiO_2_ this ratio was demonstrated as 1:4 through possession of all the other constant variables. According to several investigators [39], the change rate was significant at catalyst lesser meditation, while harvest overthrow was directly related to facilitator quantity. The maximum amount of catalysts resulted in unwanted products that slowed biodiesel yield [40]. The data in (Table 1) also revealed that TiO_2_ has a relatively higher catalytic proficiency, followed by KPC and CPC, on oil alteration into FAMEs. The reduced atom size of TiO_2_ (0.5 µm) delivers high superficiality towards volume percentage; hence, additional active spots were accessible for reactant compounds. The current study exposed the variations in FAMEs that have been synthesized through using different heterogenous nano-catalysts to keep the total of oil and all other variables continuous. The inconsistency in proportion of oil alteration into esters is the outcome of the relative competence of heterogenous nano-catalysts [16,41].

### 3.2. Representation of Heterogeneous Nano-Catalysts, CPC, KPC as Well as TiO_2_

#### 3.2.1. CPC and KPC X-ray Diffraction (XRD)

**Figure 3:**
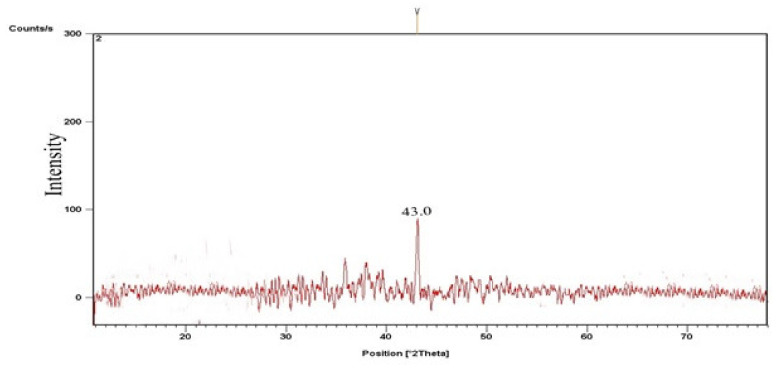
XRD results of CPC nano-catalyst.

**Figure 4:**
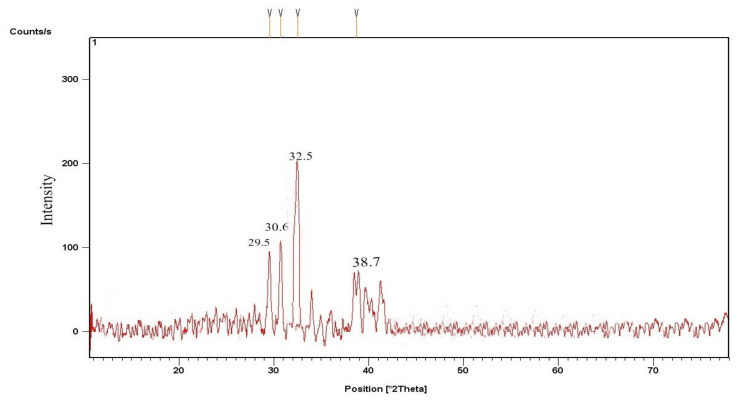
XRD results of KPC nano-catalyst.

XRD design based on CPC nano-catalysts as in (Figure 3) shows a deflection peak at 2 theta angles at 43.0, which can be perfectly filled to 200, respectively. Under optimal conditions, the XRD design of diverse nanoparticles of KPC has been exposed to prominent diffraction at 2 thetas (Figure 4), i.e., 29.5, 30.6, 32.5, 38.7, respectively. The physical analysis of CPC and KPC was resolute using XRD illustrations (Figure 3 and Figure 4) as then subsidizing the cellulose substantially. The amorphous essence of both agents referred to the desired orientation of the constituent parts in such a way to contribute very skinny peaks adjacent to each other [42]. The peaks become sharper after calcination and at 2 thetas at 29.5, demonstrating more arrangement and solidification of quartz. Likewise, another peak appeared at 2 thetas at 43.0, specifying the incidence of (quartz). These 2 thetas were also perceived by [43]. XRD pattern in (Figure 4) has revealed that the KPC was the utmost amorphous illustration. Moreover, this outcome has been confirmed by [27], who stimulate numerous unwanted agricultural materials through KOH. Furthermore, the amorphous complexion of the activated carbon has been reported previously by most scholars as well [44].

#### 3.2.2. TiO_2_ Catalyst’s X-ray Diffraction 

**Figure 5:**
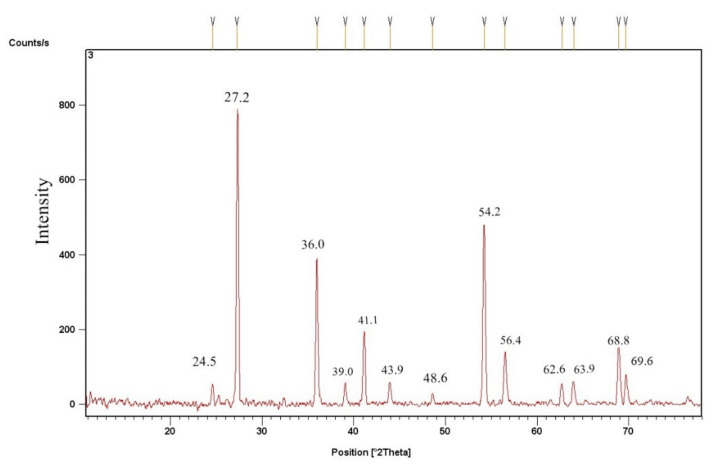
XRD results of TiO_2_ nano-catalyst.

The XRD analysis of the already-prepared TiO_2_ sample’s nanoparticles was done using a Bruker make diffractometer, comprising (Cu-Kα) X-rays with a wavelength of 0.154 nm, and data were acquired at 2θ with a range of (10°–80°). The result shows that the crystal structure of TiO_2_ is tetragonal (Anatase) and has particle sizes of 49 nm to 60 nm. The XRD design of TiO_2_ Nano-catalyst (Figure 5) indicates strong deflection heights at 2 theta angles viz. 24.5, 27.2, 36.0, 39.0, 41.1, 43.9, 48.6, 54.2, 56.4, 62.6, 63.9, 68.8, and 69.6, which can be perfectly filled to, 101, 110, 101, 200, 111, 210, 211, 220, 022, 310, 301, 112, correspondingly. The average quartz size of the TiO_2_ Nanocatalyst (35–74 nm) stood recognized with the help of the D-Scherer equation (D = Kλ/βcosθ). The 2 theta peaks at 27.2°, 36.0°, and 54.2° confirm its Anatase structure by showing strong diffraction peaks. [45]. The magnitude of the sample’s XRD points reveals that the designed nanoparticles stay crystalline, and wide-ranging diffraction peaks specify the very small extent of crystallite [28].

#### 3.2.3. SEM Findings of Nano-Catalysts

Characterization of particle size and morphology of the produced mixed nano-catalysts, HNC, the scanning descriptions were done with SEM; Jeol Model (JSM-6390LV) appliance. Different magnifications were used for SEM of nano-catalysts’ images.

##### SEM of CPC and KPC

**Figure 6:**
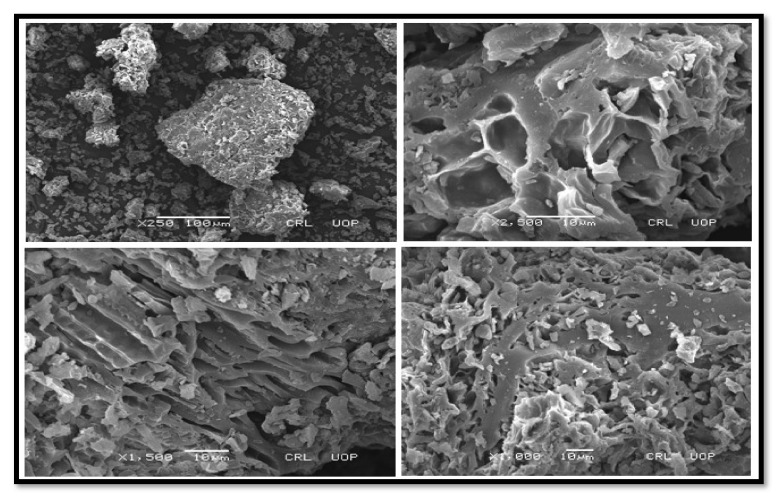
SEM images of CPC with changed magnifications.

**Figure 7:**
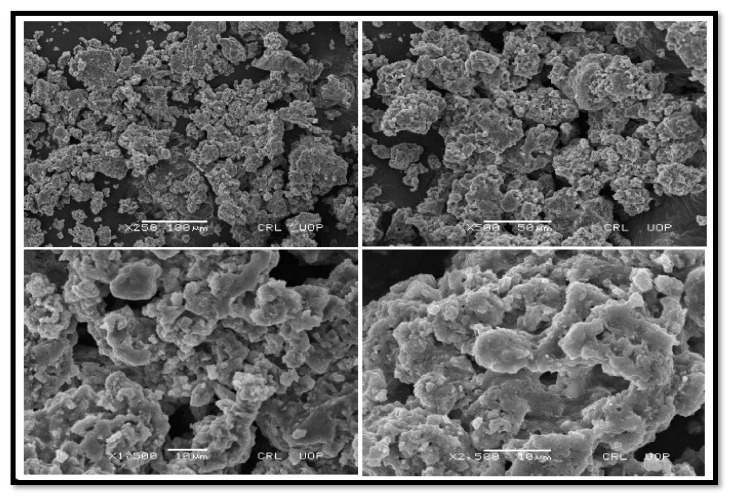
SEM images of KPC with dissimilar magnifications.

The SEM results have shown the external morphology of each substance. The surface morphology of CPC and KPC has been shown in (Figure 6 and Figure 7). CPC shows that the roughness of the surface was increased after calcination, many macropores and hollow cavities have appeared, and as a result, the external structure becomes very porous (Figure 6). These changes in morphology might boost the catalytic effectiveness of CPC as compared to non-calcined *P. aitchisonii* oil cake. *P. aitchisonii* oil cake stimulation with KOH gives rise to the KPCs surface regulating and smoothing positively (Figure 7). Numerous mesopores seemed rounded pits spread on the superficial of KPC and perform as canals. Mesopores are not desirable and not operational during the catalytic procedure. The fluffy materials and milky particles on the surface of KPC are possibly due to the existence of KOH deposits and maybe some supplementary contaminations [46].

##### SEM of TiO_2_

**Figure 8:**
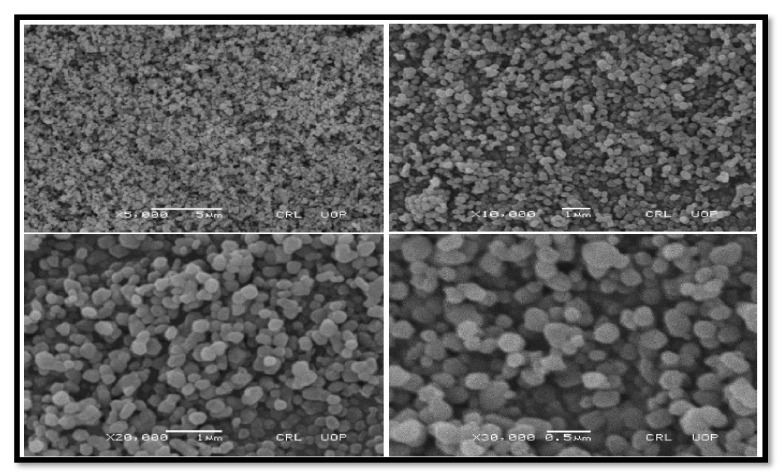
SEM images of TiO_2_ with different magnifications.

The SEM study demonstrated the development of TiO_2_ nanoparticles and their morphological dimensions in such a way that the usual size fluctuated from 50–80 nm with burying particle dimensions. The TiO_2_ nanoparticle appeared spherical (Figure 8). The larger aggregated particles of TiO_2_ remained visible because of the aggregation of smaller nanoparticles [28].

#### 3.2.4. Representation of (EDX) Analysis of CPC Heterogenous Nano-Catalyst

**Figure 9:**
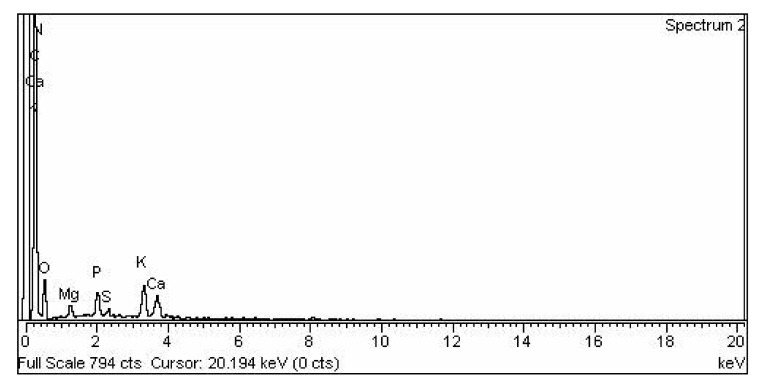
EDX analysis of CPC.

EDX analysis was performed to figure out the configuration of the mentioned catalyst. EDX has clearly shown the existence of Carbon, Nitrogen, Oxygen, Magnesium, Phosphorus, Sulphur, Potassium, and Calcium in the applied catalyst (Figure 9). This catalyst consists 60.45% of Carbon, 13.22% (Nitrogen), 20.06% (Oxygen), 0.62% (Magnesium), 1.43% (Potassium), 0.30% (Sulphur), 2.35% (Potassium), and 1.57% (Calcium) confirm the purity of CPC nanoparticles. EDX results revealed that CPC nanoparticles mainly consist of Carbon and Oxygen and are appropriate to be recycled as a catalyst (Table 2).

#### 3.2.5. KPC Heterogenous Nano-Catalyst EDX

**Figure 10:**
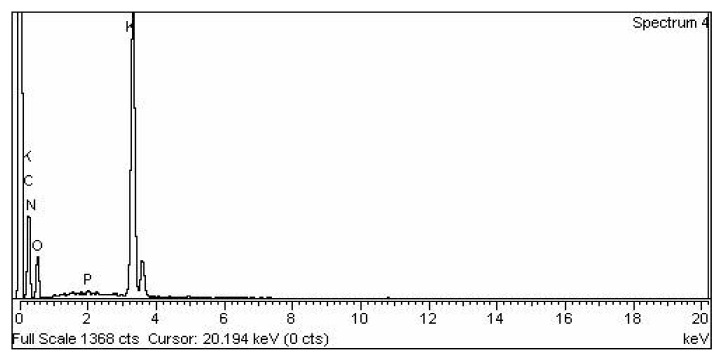
EDX analysis of KPC.

EDX has prominently shown the manifestation of Carbon, Nitrogen, Oxygen, Phosphorus, and Potassium in the KPC catalyst (Figure 10). The chemical agent is comprised of Carbon (26.05%), 4.54% Nitrogen, 34.38% Oxygen, 0.38% Phosphorus, and 34.65% Potassium, which has been shown in the pureness of KPC heterogenous nano-catalytic reagent. Results have shown that KPC heterogenous nano-catalyst mainly contains Carbon, Oxygen, and Potassium, which reveals that KPC was pertinent to be used as a catalytic agent (Table 3).

#### 3.2.6. TiO_2_ Nano-Catalyst Energy Dispersive X-ray EDX Analysis

**Figure 11:**
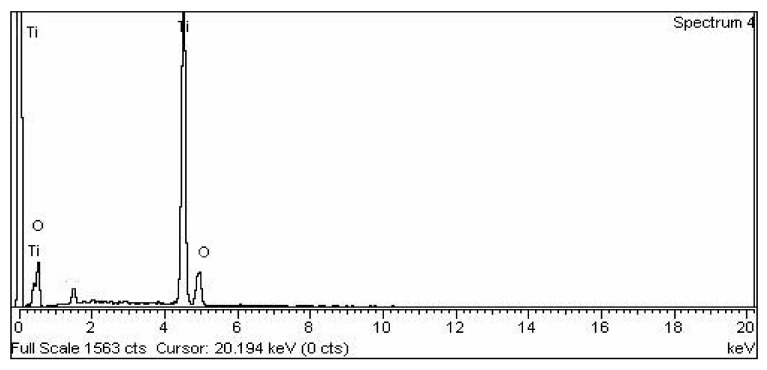
EDX analysis of TiO_2_.

The composition of the TiO_2_ catalyst has presented the presence of Oxygen and Titanium from the EDX outcomes (Figure 11). The nano-catalyst consists of 58.43% Titanium and 41.57% Oxygen, which shows the purity of TiO_2_ nanoparticles. It is obvious from the results that TiO_2_ nanoparticles were appropriate to be used as a catalyst (Table 4).

### 3.3. Characterizations of Synthesized FAMEs (Fatty Acid Methyl Esters)

#### Comparative (GC-MS) Investigation of *P. aitchisonii* Biodiesel

**Figure 12:**
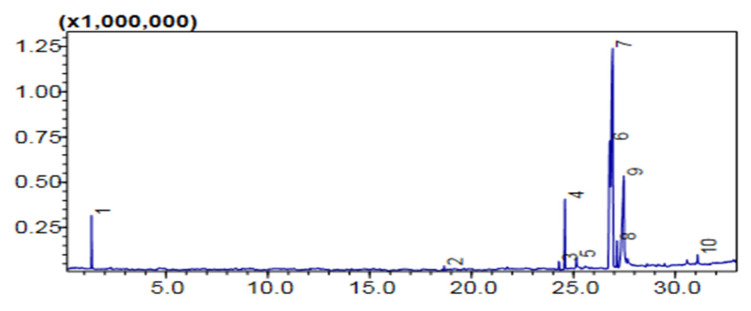
GC Chromatogram of PAOB treated with TiO_2_.

**Figure 13:**
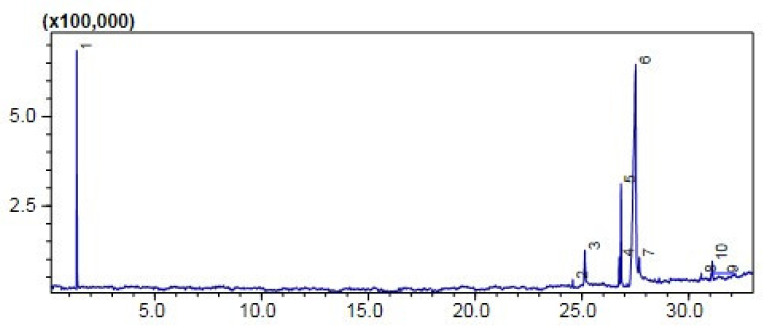
GC Chromatogram of PAOB treated with KPC.

**Figure 14:**
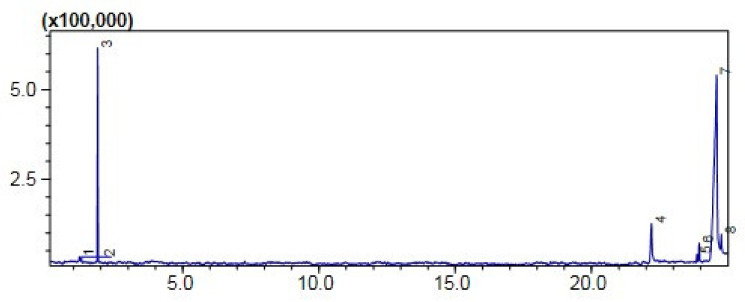
GC Chromatogram PAOB treated with CPC.

GCMS was one of the extensively and very suitable logical techniques for quantifying structure, chemical configuration, and types of FAME that exist in biofuel. However, the subsequent hierarchy illustrated the efficiency of heterogenous nano-catalysts such as TiO_2_ > KPC > CPC on the synthesis of biodiesel yield, while the methyl-esters percentage expressed by the heterogenous nano-catalysts was 74.5% > 58% > 50%, correspondingly (Table 5). The efficiency of nanocatalysts of TiO_2_ was maximized due to its precise features such as minor size particle and consequently maximized surface area. GCMS spectra of TiO_2_, CPC, and KPC catalyzed biodiesel has shown prominent (11) heights, and each height parallels to an individual FAME, which was more recognized through corresponding with NIST library 11. These eminent FAMEs might probably be Dodecanoic-acid methyl-esters, 9-Hexadecenoic acid methyl esters, Hexa-decanoic acid-methyl esters, 9-12-Octa-decadienoic acid methyl esters, 9-Octadecenoic acid methyl esters, Octadecenoic acid methyl esters, 9-Octadecenoic acid, 1,2,3-(dihydroxy propyl) ester, (9,12,15)-Octadecatrienoic acid ethyl ester, (9)-Octadecenoic acid-1,2,3 propanetriyl E, 9-Octadecenoic_acid, 2,3-dihydroxy propyl ester, Carbonochloridic acid ethyl esters. The maintenance time of all these FAMEs is demonstrated in (Table 5). The FAMEs stood recognized by organizing the holding time’s data and further confirmations were completed via inner criterions of GC-MS (Figure 12, Figure 13 and Figure 14).

### 3.4. FT-IR of P. aitchisonii Seed Oil Biodiesel (PAOB)

**Figure 15:**
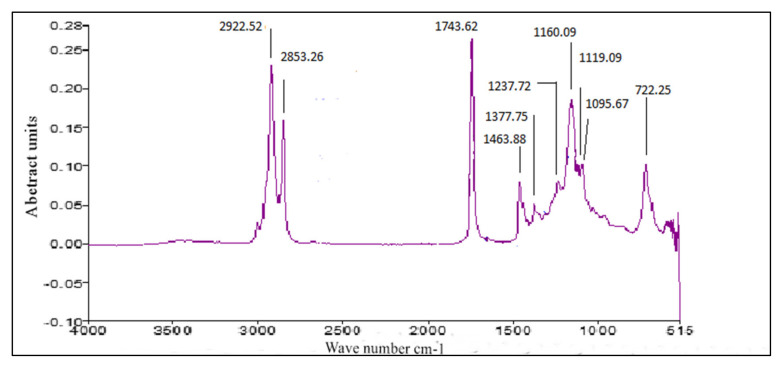
FT-IR analysis of PAOB by using CPC heterogenous nano-catalyst.

**Figure 16:**
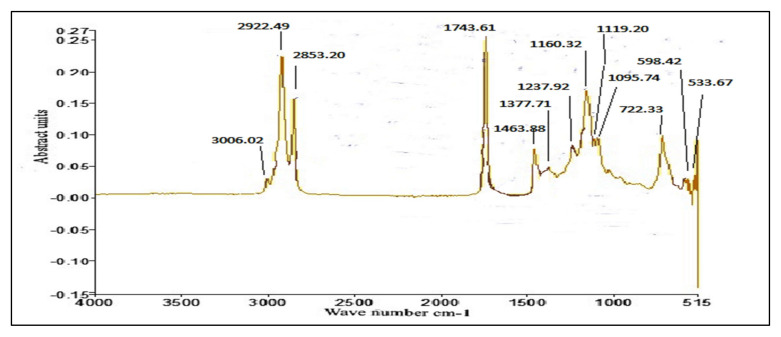
FT-IR analysis *Prunus aitchisonii* by using KPC.

**Figure 17:**
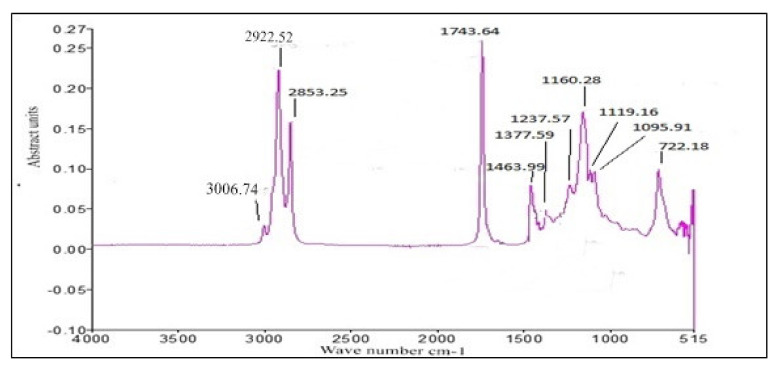
FT-IR analysis of *Prunus aitchisonii* oil biodiesels by using TiO_2_ nano-catalyst.

FT-IR is faster, and an easier analytical practice used for the monitoring of FAMEs synthesis during the trans-esterification reaction, recognition of functional groups, and chemical bonds in a certain chemical compound. Bending and stretching of a bond that is present or absent in a specific functional group is demonstrated through FT-IR. Figure 15, Figure 16 and Figure 17 displays that the existence of C-Br stretch Alkyl halides, Aliphatic amines and Aromatics existed, such as was specified after medium-plus-strong peaks of carbon and C-N stretch. The solid peak displayed a C-O bond stretch that highlights the existence of Ester, Carboxylic-acid, Alcohols, and Ethers. The double mediocre peaks of 1463.99 cm^−1^ and 722.25 (cm^−1^) demonstrate the presence of Alkanes. FT-IR spectra show evidence for very strong peaks of 1743.64 cm^−1^ besides 1095.91 cm^−1^ and show the -C=O stretch as well as C-O stretch that are the evidence of aliphatic esters (esters, carboxylic acid). A strong height of 2922.52 cm^−1^ is an illustration of the existence of O-H stretch. Furthermore, the point at 3006.74 cm^−1^ spots the evidence of aromatic compounds. FT-IR spectra reveal substantiation for the existence of numerous compounds in the biodiesel of *P. aitchisonii*, which includes a strong peak of 1743.64 cm^−1^ as ester group that is an obvious confirmation. 

#### Comparative Analysis of FT-IR of *P. aitchisonii* FAMEs

*P. aitchisonii* seed oil conversion into resultant FAMEs by the process of transesterification can be demonstrated in the response turns through observing the variations in assured functional groups portrayed on the FT-IR spectrum of *P. aitchisonii* FAME. The FT-IR spectra’s comparison of the catalysts TiO_2_, CPC, and KPC shows the existence of a pure illustration of the alteration their particular methyl-esters (Figure 15, Figure 16 and Figure 17). While comparing the results, it is illustrated that alkanes were present in all the three samples. The existence of the alkyl halide (C-Br) group in the KPC sample was indicated, but no stretch happens in the instance of CPC and TiO_2_. In all the three samples’ spectra, C-O stretch pointed out the presence of carboxylic acid, esters, ether, alcohols and. However, the aliphatic amines are indicated in all the samples as shown in the strong heights in the range of 1237.57 cm^−1^. The occurrence of alcohol and phenol are underlined through definite heights, whereas no such verification is found in the incident of all samples because of a lack of -OH stretch. However, the C-O elasticity exists, which specifies any kind of alcohol, carboxylic acid, ester, and ether. The solid peak was 1377.59 cm^−1^ with the appearance of acetyl-acetonates and a solid peak of 1119.16 cm^−1^ is symbolic of cyclopentadienyls, compounds present in all three samples. The strong peak of C-H stretch was 3006.74 cm^−1^ indicates the presence of aromatic compounds present in KPC and TiO_2,_ but no such stretch occurs in the CPC-spectrum. The presence of a medium-sized peak of 2922.52 cm^−1^ illustrates the presence of alkanes. The presence of a strong stretch of C=O (1743.62 cm^−1^) in all three catalysts indicated the manifestations of esters and saturated aliphatic compounds. 

### 3.5. Comparative NMR Analysis of Prunus aitchisonii FAMEs

#### 3.5.1. H NMR Assessment of *P. aitchisonii* Biodiesel Deal with TiO_2_

**Figure 18:**
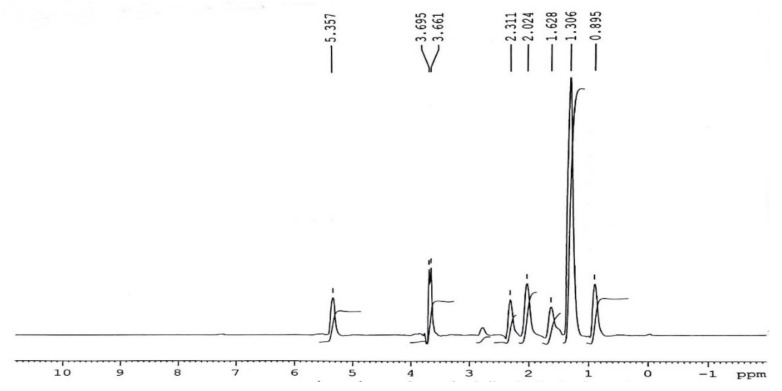
^1^H-NMR spectrum of *Prunus aitchisonii* biodiesel treated with TiO_2_.

The distinguishing topmost of methoxy proton was noticed as a single line on 3.661–3.695 ppm as well as of Alpha-CH_2_ protons on 2.31 ppm (Figure 18). Both of these are the distinctive peaks for the verification of methyl esters existent in biodiesel. Other points are observed at the end of methyl protons at 0.895 ppm. The sturdy indication was next to 1.306 ppm correlated to methylene-protons of carbon series. The additional point seemed at 1.628 ppm on account of Beta-carbonyl methylene proton as well as on 5.357 ppm owing to olefin (hydrogen) [47,48]. ^1^H-NMR could be operated furthermore to compute the transformation of seed oils within methyl-esters as a result of trans-esterification reactions [49,50]. The related indication selected for incorporation were both of methoxy-group amongst methyl-esters at 3.62 ppm and also α-carbonyl methylene protons at 2.25 ppm.

Calculations that are applied for measuring the product of trans-esterification are cited as equation number (1). The ratio of alteration of triglycerides into consistent methyl esters via such comparison is created as 93.3% ± 2 and that is relatively good in arrangement almost along the practical yield, showing 96.5% over equation number (3). 

The less useful income as linked to intended yield through ^1^HNMR is perhaps enhanced via granting an extra relaxing phase to the invention, employing an assortment of more effective isolation procedures, such as centrifugation (Figure 18).

#### 3.5.2. C-NMR Examination of *P. aitchisonii* FAME Deals with (TiO_2_)

**Figure 19:**
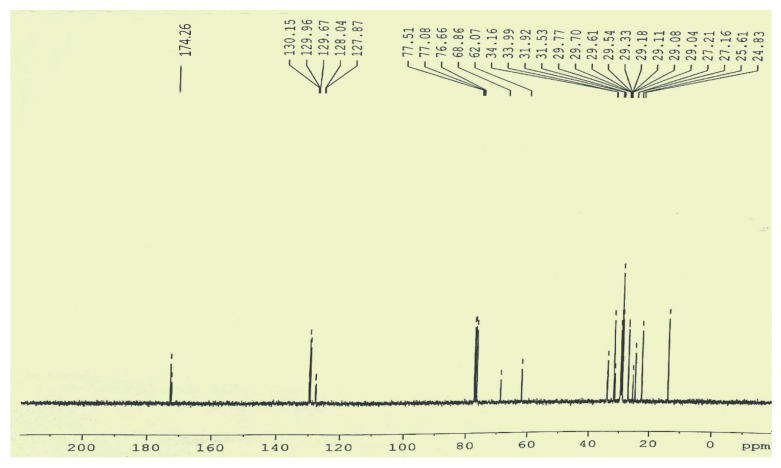
^13^C NMR spectrum of *Prunus aitchisonii* biodiesel treated with TiO_2_.

Most likely in the ^13^C NMR range, variations have been observed (Figure 19), the majority of those points falling in the assortment of 24.83–34.16 ppm equivalent to the CH_2_, CH_3_ in addition to allylic carbon atom. Indications on 174.26 ppm and 130.15 ppm were agreed towards carbonyl (-C=O) carbon particles of the tri-esters functional grouping as well as un-saturation between methyl esters. Signs at 128.04 ppm were consigned toward vinylic (C=H), however, the signals existing at (24.83–29.77 ppm) are allocated to the terminal (CH_3_) “carbon” atom of the fatty-acids chain. The indication lines at (34.16 ppm) matched with (CH_2_ carbon) atom of a similar chain. The absence of signal lines at “172.75–173.16 ppm” approved the occurrence of (C=O) carbon atom (Figure 19).

#### 3.5.3. “1. H NMR” Analysis of *P. aitchisonii* FAMEs Resulted from CPC

**Figure 20:**
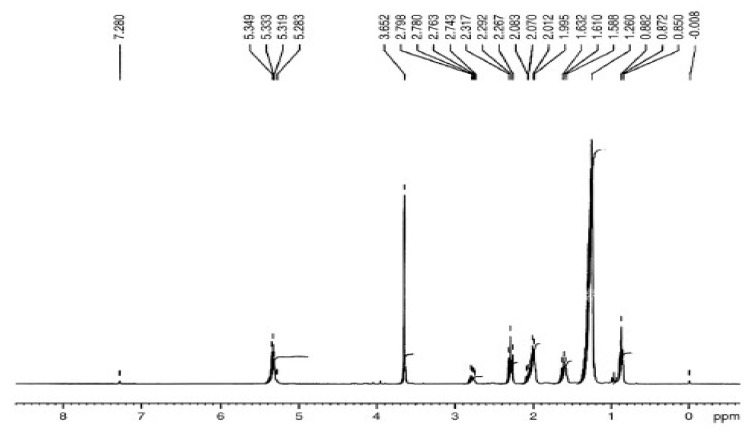
^1^H NMR spectrum of *Prunus aitchisonii* biodiesel treated with CPC.

The ^1^H NMR spectrum of *P. aitchisonii* treated with CPC displayed in Figure 20 presenting the typical single peak of methoxy protons at 3.65 ppm as well as a triplet belonging to α-CH_2_ protons at 2.26 ppm. These distinctive dual peaks are the validation of methyl esters in biodiesel. Terminal CH_3_ proton peak at 0.85 ppm besides the extreme signal of methylene protons of the long chain of esters at 1.26 ppm were observed. At 1.58 ppm, the point is related to Beta-carbonyl (methylene) proton then at “5.28 ppm” in line for olefin’s protons. By computing, the result of percentage exchange of triglycerides was found as 90.5 ±2%, which is reasonably in virtuous assurance almost with a perceived result such as 92.58% through Equation (1) (Figure 20). 

#### 3.5.4. C NMR Analysis of *P. aitchisonii* FAMEs Treated with CPC

**Figure 21:**
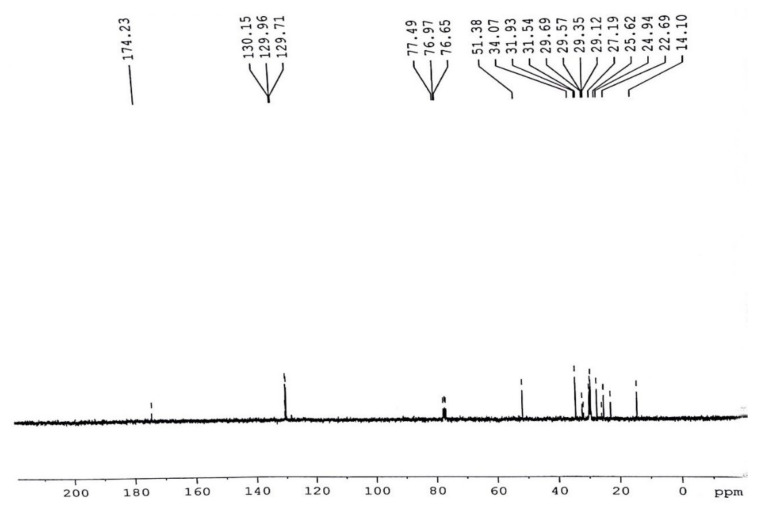
^13^C NMR spectrum of *Prunus aitchisonii* seed oil biodiesel treated with CPC.

The ^13^C NMR spectrum is used to analyze the structural characterization of *P. aitchisonii* FAMEs. As anticipated in ^13^C NMR continuum, various signals were comprehended in (Figure 21). Analogous to CH_3_, CH_2_ also allylic carbon atom, most of the pointers happened in the array of (14.10–34.07 ppm), respectively. The signals at 174.23 and 51.38 ppm are the characteristic peaks of the ester carbonyl group (-COO-) and C-O (methoxy carbon) respectively. The peaks that appeared at (130.15) and (129.71) ppm indicated the “unsaturation” in methyl esters of *P. aitchisonii*. In the equivalent sequence of frequency absorption, supplementary peaks are related to methylene and ethylene carbon of long carbon chain in (FAMEs) fatty acid methyl esters (Figure 21).

#### 3.5.5. H NMR Analysis of *P. aitchisonii* FAMEs Treated with KPC

**Figure 22:**
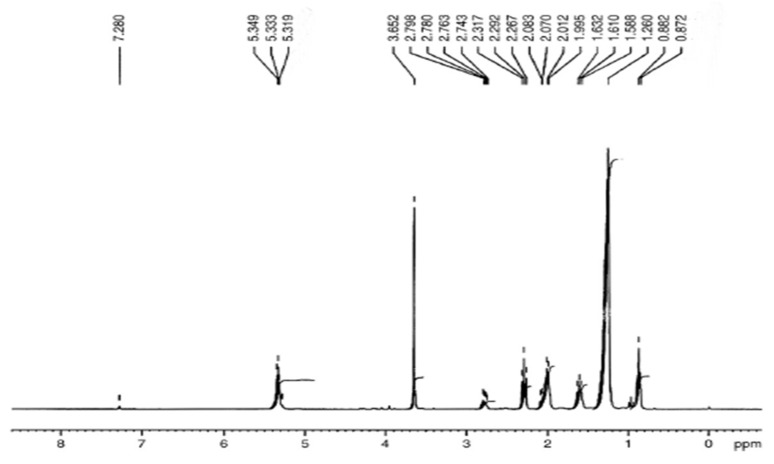
^1^H NMR spectrum of *Prunus aitchisonii* oil biodiesel treated with KPC.

The ^1^H-NMR band of *P. aitchisonii* seed oil treated with KPC is given in Figure 22. The triplet at 5.319–5.349 ppm illustrated the olefinic protons. At 3.652 ppm, the conspicuous singlet is expressive methoxy protons of esters functional group in biodiesel. The multiplet at 2.74–2.79 ppm specifies the “bis” allylic protons of the unsaturated fatty acid chain. For 2.26–2.31 ppm, a triplet represents an Alpha-methylene proton of esters. The α- methylene protons of ester and Beta- methylene protons in ester both appeared as a multiplet at 1.99–2.08 ppm and 1.58–1.63 ppm, individually. Terminal (methyl) protons at 0.87–0.88 ppm appeared as a doublet. The singlet at 3.65 ppm specifies the ester’s methoxy-protons, which was the indication of the alteration of PASO into PAOB. The ^1^H NMR spectrum is used to examine biodiesel and fatty acid arrangement through using the zones of the indications of methoxy as well as methylene protons to display the product of transesterification [51,52]. The peaks which may tolerate the evaluation of unsaturated FAMES were those of the protons of olefinic (5.31–5.34 ppm), bisallylic carbons (2.74–2.79 ppm), Allylic-carbons (2.0–2.2 ppm), and terminal methyl-groups (0.87–0.88 ppm). The saturated “FAMEs” can be resolute via the indication of methylene, CH_2_—protons, 1.2–1.6 ppm. The fraction alteration of “triglycerides” to consistent biodiesel with Equation (1) was found (92.4 ± 2.5%), which is relatively nearby to the nearly practical yield of 94.5% through Equation (1) (Figure 22).

#### 3.5.6. C NMR Analysis of *Prunus aitchisonii* Biodiesel Treated with KPC

**Figure 23:**
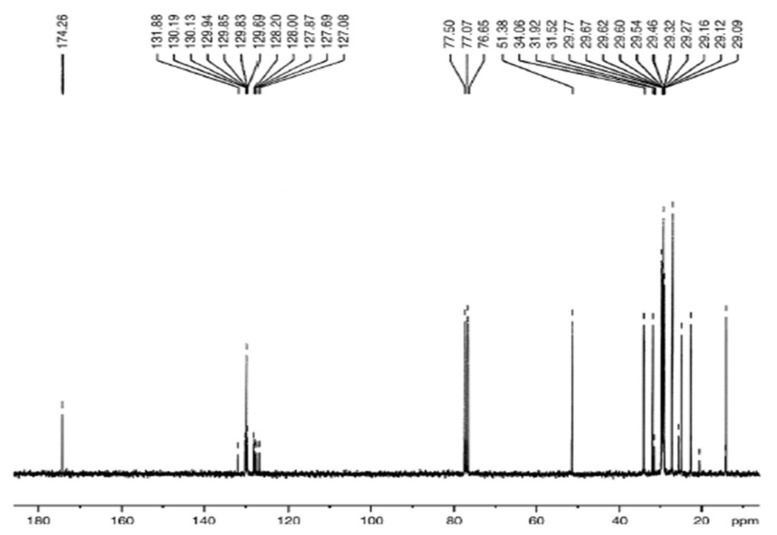
^13^C NMR spectrum of *Prunus aitchisonii* seed oil biodiesel treated with KPC.

The descriptive scale of ^13^C NMR of FAMEs illustrated in Figure 23 has shown the representative peaks of carbonyl esters -COO- plus C-O at 174.26 ppm and 51.38 ppm, separately. The heights about 131.88 ppm and 127.08 ppm specified the un-saturation in methyl-esters. Further peaks at 14 ppm were in line for terminal carbon of CH_3_ groups and signals at 22.09–34.06 ppm are associated with “methylene” carbons of extended carbon sequence in FAMEs (Figure 23).

### 3.6. Biodiesel Optimization

Successive trials were performed under predesigned conditions to obtain maximum biodiesel yield. The following four different variables were assigned for this purpose to demonstrate their special effects on the FAMEs result.

1. Oil and methanol proportion;
2. Amount of catalysts;
3. The temperature of the chemical reaction;
4. The time period of reaction.

#### 3.6.1. Oil and Methanol Proportion

The production rate of biodiesel was affected prominently by the most active variable, the proportion of oil and methanol [53]. In previous study, maximum methyl esters conversions were obtained from corn oil from a 1:6 ratio of oil to methanol [54]. Results of the matching ratio were also determined by another scientist while using seed oil of the cotton plant for biodiesel production [55], and also using rocket seed oil for transesterification reactions [56]. When the ratio of oil to methanol was kept at more than 1 to 3 then this also caused an increment in the amount of FAMEs during transesterification reaction; however, the maximum volume of methanol correspondingly resulted in the solubility of glycerol, generating difficulties in sorting out of glycerol and FAMEs [57].

**Figure 24:**
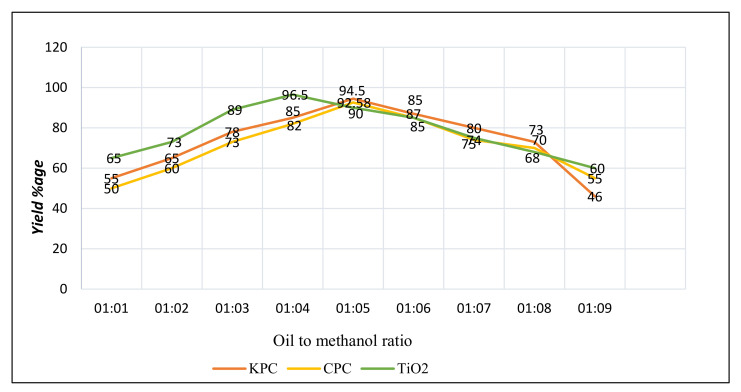
Oil-to-methanol ratio effect on transesterification, using 3 different types of catalysts while other variables were kept constant.

The relationship of oil and methanol contrasts was observed in ratios of 1:3, 1:4, 1:5, 1:6, and 1:7 in the current study by keeping additional variables constant such as catalyst concentration, temperature of reaction, and reaction time period. The extreme biodiesel’s yield was recorded on a 1:5 ratio with CPC and KPC, but in the case of TiO2, this ratio was 1:4 for the maximum biodiesel yield (Figure 24). In case of CPC and KPC, the biodiesel production was decreasing at 1:6 and 1:4 oil-to-methanol ratio, but in case of TiO2, the biodiesel yield decrement occurred at 1:5 and 1:3 oil-to-methanol ratio, which was perhaps due to solubility of glycerin in fatty acid methyl esters (FAMEs). 

#### 3.6.2. Amount of Catalysts

**Figure 25:**
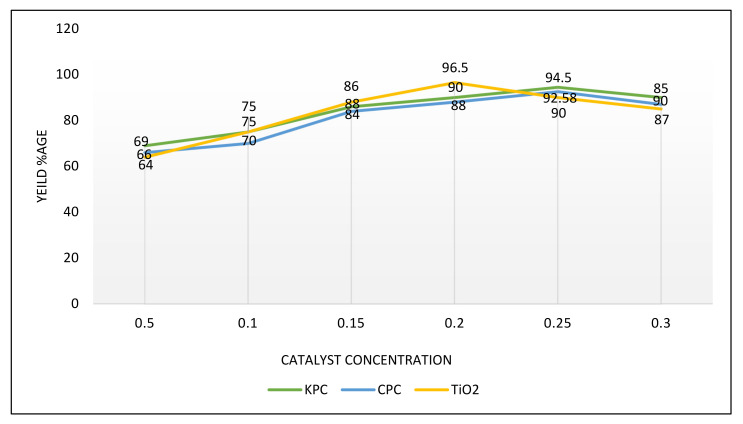
Level of catalyst effect on transesterification, while other variables were kept constant (temperature, time period, oil-to-methanol ratio).

At a smaller amount of oil and methanol percentage, FAMEs concentration increased as per the concentration of the catalyst rose [58]. From the literature, it was found that the production of FAMEs decreased during transesterification reaction when catalyst concentration increase in range 0.5–1.0% [59]. The catalyst amounts used in transesterification reaction were 0.5, 0.10, 0.15, 0.20, 0.25 gm. To obtain maximum biodiesel output, other variables were kept continuous, i.e., oil-to-methanol ratio, time period of the reaction, and temperature of the reaction (Figure 25).

In the current study, the result shows that as the catalyst concentration increases up to 0.3 gm, then the biodiesel yield decreases, respectively, as given in Figure 25. The concentration below 0.3 gm is suitable for the maximum quantity of biodiesel. On the application of CPC and KPC the maximum yield of biodiesel obtained at 0.25 gm while in the case of TiO2, the maximal yield of biodiesel is attained at 0.20 gm. 

#### 3.6.3. Temperature of Reaction

The elevated temperature will hasten the “transesterification” reaction level at a certain range. The fact is found in many experimental studies that the finest results of transesterification reactions were obtained at 60–70 °C temperature range [60]. Another study deliberated the influence of temperature on FAMEs yield by using edible and nonedible vegetable oils. The reaction temperature was set in the 40–120 °C range and the passionate conclusion of “canola” oil was observed at “60 °C” [58].

**Figure 26:**
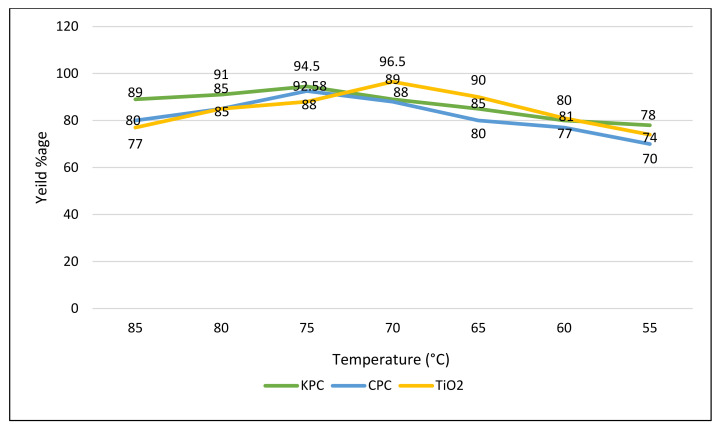
Temperature effect on transesterification, while other variables (catalyst amount, time period) were kept constant.

In present study, the reaction heat was modified at 50, 55, 60, 65, 70, and 75 °C to analyze its influence on the product (biodiesel), while additional variables were kept constant, i.e., oil-to-methanol ratio, time period of reaction and catalytic agent concentration (Figure 26). In the case of CPC and KPC, the best results were observed at 70 °C, while in the case of TiO_2_ nano-catalyst the best results were observed at 75 °C. At a certain range, the biodiesel amount increases with the rise in temperature of the transesterification reaction (Figure 26).

#### 3.6.4. Time Period of Reaction

**Figure 27:**
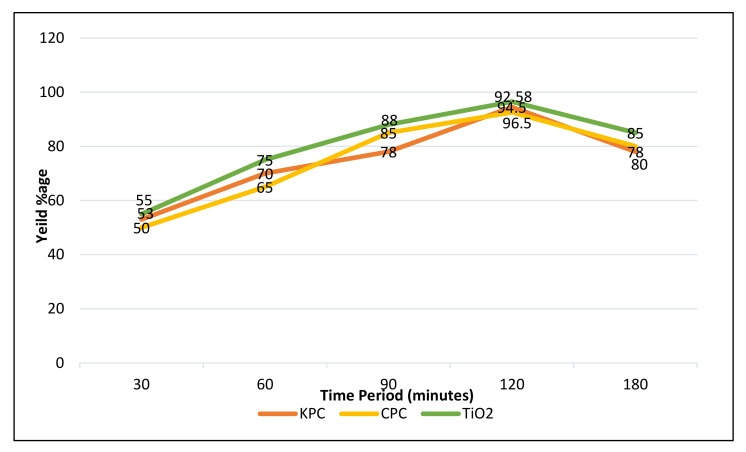
Time period effect on transesterification, while other variables were kept constant (temperature, catalyst amount, oil-to-methanol ratio).

For the illustration of chemical reaction time influence on the yield of biodiesel production, consignment experiments were conducted through keeping the other variables constant, i.e., oil-to-methanol ratio, catalyst concentration, temperature of reaction for the different reaction time periods such as 30, 60, 90, 120, and 180 min for all the three different catalysts (Figure 27). It is quite obvious from the information that in general biodiesel yield product rises with the increases in the time period of the reaction up to 120 min for all three heterogenous nano-catalysts (HCNs). Furthermore, PASO has higher amplification into biodiesel production with all the three catalysts, respectively. 

### 3.7. Physical Properties of P. aitchisonii Oil Biodiesel (PAOB)

PAOB fuel properties were determined quantitatively as well as matched using the standards for biodiesel from the American society for testing and materials (ASTM). These properties include “Color”, “Flashpoint” °C, “Density” @ of 15 °C kg/L, “Kinematic viscosity” @ of 40 °C, “Pour point” °C, “Cloud point” °C, “Sulphur” % wt and “Total Acid No”, mg KOH/gm were measured in accordance with ASTM D-1500, ASTM D-93, ASTM D-1298, ASTM D-445, ASTM D-97, ASTM D-2500, ASTM D-4294, and ASTM D-974, individually.

#### 3.7.1. Flash-Point

Flashpoint is the particular temperature upon which a biodiesel has ignition. It involves the measurement of substance affinity in which a combustible mixture is formed in the occurrence of air [59]. This point is a component of applied impact. During storage, handling, and transportation, a high flash point is safe [61]. According to the scientist in Bangladesh, the rubber seed oil flashpoint was observed at 120 °C [62]. This physical property of *Pongamia pinnata* was demonstrated as a result of such temperatures as 150 °C by using the ASTM D-93, testing method [63]. In the present research, the flashpoint of PAOB was documented as “71.5 °C” (Table 6), which comes with in the standard array of ASTM D_93 which reveals that this PAOB is riskless “fuel”. The flashpoint of high-geared diesel falls in the range of 60 to 80 °C. 

#### 3.7.2. Density

Significantly, during the identification of biodiesel reputation, density plays a vital role [64]. It is observed that denser oil biodiesel has more energy [65]. According to the previous literature, sesame oil biodiesel has 0.871 kg/L density [66]. Similarly, it was documented that ‘rice bran’ oil biodiesel has a density of (0.877) kg/L [59]. Almost the same kind of results was found in other studies [53], that the oil biodiesel of *Sinapis alba* has a density value of (0.872 kg/L). In the current research, the density of PAOB is resulted as (0.836) @ 15 °C, (Table 6), while the density of (high-speed) diesel is (0.834). It is illustrated from the results that PAOB has almost equal density to that of ‘high-speed’ diesel. 

#### 3.7.3. Kinematic Viscosity

Kinematic viscosity is the adhesiveness of fuel or biodiesel, which has shown by the index [67]. The reduced value of diesel viscosity can hinder or prevent the engine lubrication effects, so the biodiesel kinematic would be established agreeing with the standards of ASTM. According to Murshid et al. (2011), it was documented that the oil biofuel of rubber has kinematic viscidness of 4.5 at 40 °C, which stood higher than the viscosity of ‘high’ speed diesel at “4.223” [68]. The same results for sesame seed oil biodiesel with 5.77 kinematic viscosity were also found out in [66]. In the same way, the *Sinapis alba* seed biodiesel’s viscosity was predetermined as 5.45, which is also greater than the standards of ASTM. The cause of high viscidness of oil biodiesel is the presence of greater molecular mass compounds with their immense chemical configurations. The kinematic viscosity of PAOB was found to be 4.33 kg/L, Table 6, which lies within the standard values of ASTM and this value is almost the same but a little bit greater than high-speed diesel’s value, which is 4.22 kg/L. 

#### 3.7.4. Pour Point and Cloud Point

Among fuel’s significant properties, the top two are poured point and cloud point. Pour point is the bottom-most temperature, at which the fuel is able to run in ice-cold conditions, while the cloud point is the temperature at which paraffin becomes crystallized and starts to detach when the biofuel is chilled under recommended situations. According to Saka and Isayama, biodiesel was prepared without catalysts and the pour point value of biodiesel was resolved as −16, which fell within the high-speed diesel range, i.e., −15 and −16 [69]. It was also documented that rice fiber’s biodiesel has a pour and cloud point value of −7 and −6, individually [59]. Dessouky found that the cloud point and pour point values of corn oil biodiesel are −2 and −16 [31,60]. The values of both the pour point and cloud point of rocket’s seed oil biodiesel were also revealed, as −3 and −15, respectively [70]. Currently, the pour point and cloud point of PAOB are found as −7 and −8, Table 6, falling within the series of ASTM standards. 

#### 3.7.5. Sulphur Weight %

After treatment with H_2_SO_4_, the biodiesel is burned and ignited the biodiesel, so that we can find out the sulfur weight %. Commonly, a low sulfur weight number is perfect for areas that are highly polluted. According to the previous literature, it is observed that Pongamia oil biodiesel has a sulfur weight % of about (0.005), which was quite less in comparison to the standards of ASTM. The same results were also found out by Ahmad et al. (2013) and [63,71]. It was investigated that the sulfur weight % of rocket oil biodiesel was found to be 0.05%, and it was also reported in another study that sulfur weight % was (0.02%) in muskmelon oil biodiesel [66,72]. Ahmad et al. (2011) have stated that the sulfur content in sesame oil biodiesel is 0.01% [51]. It was also reported that the sulfur quantity in corn oil biodiesel is 0.01% [60]. The sulfur weight % of PAOB was found to be 0.00015%, Table 6, which is according to the ASTM D-4294 standards. Finally, from the current results, it is stated that biodiesel is superior to that of high-speed diesel due to the low sulfur weight %. 

#### 3.7.6. Acid-Value

The acid value is the amount of free fatty acids (FFAs) that a sample of fuel contains. For neutralizing one gram of FAMEs, it is expressed in mg KOH/gm. The high number of acid values is not favorable for the efficiency of the engine. Following the literature, oil biodiesel’s maximum acid value has been set by standards of ASTM D-664 as 0.5 KOH/mg. The acid values of *Sinapis alba* and cotton seeds oil biodiesel are 0.242 mg KOH/gm and 0.16 mg KOH/gm [53,73]. It was also found that the acid value of muskmelon oil biodiesel is 0.45 mg KOH/gm [74]. As a result, in the current study, the acid value of PAOB has been found as 0.114, KOH/gm, Table 6, which is too low to be very appropriate for use. 

## 4. Conclusions

The present study resolved some inimitable findings in two different aspects. Firstly, it presented the investigation and exploration of an original nonedible feedstock of *Prunus aitchisonii* for the first time in the list of non-edible feedstocks. Secondly, it undertook a reasonable analysis of three different heterogenous nano-catalysts on the making of biodiesel from *Prunus aitchisonii* seed oil (PASO). The three different heterogenous nano-catalysts, i.e., TiO_2_, KPC, and CPC were made-up chemo-mechanically. For biodiesel production, these catalysts were characterized and applied to *Prunus aitchisonii* seed oil (PASO). Amongst totally used catalysts, TiO_2_ displayed encouraging results, i.e., uppermost change efficiency was accomplished up to 96.5% at 0.20 gm catalyst loading and improved experimental variables were considered as an oil-to-methanol ratio of 1:4, a temperature of 75-degree Celsius, and a response time of 120 min using reflux transesterification reaction, followed by KPC and CPC. The sequence of biodiesel alteration rate was in the manner of TiO_2_ > KPC > CPC. The optimized variables for the KPC and CPC were: oil-to-methanol ratio 1:5, temperature 70 °C, reaction time 120 min, and 0.25 gm catalyst amount using reflux transesterification reaction. By comparing the results, PASO was more well-suited to KPC and CPC, producing higher biodiesel yields of 94.5% and 92.58%, respectively. It may be contemplated that CPC is an optimal catalyst because of its reduced cost of preparation, shorter preparation time as compared to KPC, and by giving an approximate yield of biodiesel production (92.58%) comparable to that produced by KPC (94.5%) and better results of reusability than KPC. This current study displayed the probability of producing efficient, economical, and reusable heterogeneous catalyst CPC and viable oil source PASO from *Prunus aitchisonii* seeds for the synthesis of biodiesel with mild effective conditions, which helps in finding out methods to lower the overall biodiesel production expenditures.

## Figures and Tables

**Table 1:** Influence of three different catalysts on biodiesel “yield” of *Prunus aitchisonii* seed oil (PASO).

S. No	Name of Catalyst	Amount of Catalysts (gm)	Oil to Methanol Ratios	Time Period (h)	Temperature (°C)	Conversion %Age
1	CPC	0.25	1:5	2	70	92.58
2	KPC	0.25	1:5	2	70	94.5
3	TiO_2_	0.20	1:4	2	75	96.5

**Table 2:** Elemental conformation of CPC heterogeneous catalyst.

S. No	Element	Weight %	Atomic ta%
1	C K	60.45	67.91
2	N K	13.22	12.74
3	O K	20.06	16.92
4	Mg K	0.62	0.35
5	P K	1.43	0.62
6	S K	0.30	0.13
7	K K	2.35	0.81
8	Ca K	1.57	0.53
9	Total	100.00	100.00

**Table 3:** Chemical composition of KPC heterogenous nano-catalyst.

S. No	Element	Weight %	Atomic %
1	C K	26.05	39.15
2	N K	4.54	5.85
3	O K	34.38	38.79
4	P K	0.38	0.22
5	K K	34.65	16.00
6	Total	100.00	100.00

**Table 4:** TiO_2_ nano-catalyst’s chemical composition.

S. No	Element	Weight %	Atomic %
1	O K	41.57	67.61
2	Ti K	58.43	32.39
3	Total	100.00	100.00

**Table 5:** Comparative analysis of *Prunus aitchisonii* FAMEs (fatty acid methyl ester) using heterogenous nano-catalysts.

Heterogenous Nano-Catalysts	Peak #	Possible Compounds	Retention Time (min)	Formula	Molecular Weight g/mole	Base Peak	Percentage %	Total Percentage of Methyl Ester
**TiO_2_**	2	Dodecanoic acid methyl ester	18.658	C_13_H_26_O_2_	214	74	0.78	74.5%
	3	9-Hexadecenoic acid methyl ester	24.291	C_17_H_32_O_2_	268	55	1.33	
	4	(Hexadecanoic acid methyl ester	24.579	C_17_H_34_O_2_	270	74	11.42	
	6	9,12-Octadecadienoic acid methyl ester	26.783	C_19_H_34_O_2_	294	67	20.70	
	7	9-Octadecenoic acid methyl ester	26.928	C_19_H_36_O_2_	296	55	35.56	
	8	Octadecenoic acid methyl ester	27.139	C_19_H_38_O_2_	298	74	4.36	
	10	9-Octadecenoic acid, 1,2,3-dihydroxypropyl ester	31.094	C_21_H_40_O_4_	356	129	1.40	
**KPC**	2	Hexadecanoic acid methyl ester	24.570	C_17_H_34_O_2_	270	74	1.17	58%
	4	9,12-Octadecadienoic acid methyl ester	26.741	C_19_H_34_O_2_	294	67	4.32	
	5	9-Octadecenoic acid methyl ester	26.826	C_19_H_36_O_2_	296	55	15.05	
	6	9,12,15-Octadecatrienoic acid ethyl ester-	27.515	C_20_H_34_O_2_	306	67	31.68	
	8	9-Octadecenoic acid 1,2,3 propanetriyl ester	30.587	C_57_H_104_O_6_	884	55	0.97	
	10	9-Octadecenoic acid, 2,3-dihydroxypropyl ester	31.102	C_21_H_40_O_4_	356	129	2.86	
**CPC**	3	Carbonochloridic acid ethyl ester	1.884	C_3_H_5_ClO	108	45	43.78	50%
	5	9-12-Octadecadienoic acid methyl esters	23.867	C_19_H_34_O_2_	294	73	1.72	
	6	9-Octadecenoic acid methyl esters	23.949	C_19_H_36_O_2_	296	67	3.78	

**Table 6:** Fuel properties of *Prunus aitchisonii* oil biodiesel (PAOB).

S. No	Fuel Properties	Testing Methods	ASTM Standards	Results
1	Color	ASTM D-1500	2	Visual
2	Flashpoint (°C)	ASTM D-93	60–100	71.5
3	Density @ 15 °C	ASTM D-1298	0.86–0.90	0.836
4	Kinematic viscosity @ 40 °C	ASTM D-445	1.9–6.0	4.33
5	Pour point °C	ASTM D-97	−15 to 16	−7
6	Cloud point °C	ASTM D-2500	−3 to 12	−8
7	Sulfur % wt	ASTM D-4294	0.05	0.00015
8	Total acid number mg KOH/gm	ASTM D-974	0.5	0.114

## Data Availability

Not applicable.
